# Dietary compounds and cutaneous malignant melanoma: recent advances from a biological perspective

**DOI:** 10.1186/s12986-019-0365-4

**Published:** 2019-05-21

**Authors:** Maria Neve Ombra, Panagiotis Paliogiannis, Luigia Stefania Stucci, Maria Colombino, Milena Casula, Maria Cristina Sini, Antonella Manca, Grazia Palomba, Ignazio Stanganelli, Mario Mandalà, Sara Gandini, Amelia Lissia, Valentina Doneddu, Antonio Cossu, Giuseppe Palmieri, for the Italian Melanoma Intergroup (IMI), Gerardo Botti, Corrado Caracò, Annamaria Di Giacomo, Pietro Quaglino, Paola Queirolo

**Affiliations:** 1 Institute of Food Sciences, National Research Council, Avellino, Italy; 2 Department of Medical, Surgical and Experimental Sciences, University of Sassari, Viale San Pietro 43, 07100 Sassari, Italy; 3 Department of Biomedical Sciences and Human Oncology, University of Bari ‘Aldo Moro’, Bari, Italy; 4 Institute of Biomolecular Chemistry, National Research Council, Sassari, Italy; 5 Istituto Scientifico Romagnolo per Studio e Cura Tumori (IRST-IRCCS), Meldola, Italy; 6 Medical Oncology, “Papa Giovanni XXIII” Hospital, Bergamo, Italy; 7 Division of Epidemiology and Biostatistics, European Institute of Oncology, Milan, Italy

**Keywords:** Skin, Melanoma, Nutrition, Food, Dietary supplements

## Abstract

Cutaneous malignant melanoma is a heterogeneous disease, being the consequence of specific genetic alterations along several molecular pathways. Despite the increased knowledge about the biology and pathogenesis of melanoma, the incidence has grown markedly worldwide, making it extremely important to develop preventive measures. The beneficial role of correct nutrition and of some natural dietary compounds in preventing malignant melanoma has been widely demonstrated. This led to numerous studies investigating the role of several dietary attitudes, patterns, and supplements in the prevention of melanoma, and ongoing research investigates their impact in the clinical management and outcomes of patients diagnosed with the disease. This article is an overview of recent scientific advances regarding specific dietary compounds and their impact on melanoma development and treatment.

## Introduction

Nutrition plays an important role in cancer. The American Institute for Cancer Research and the World Cancer Research Fund have estimated that 30–40% of all cancers can be prevented by a proper diet, physical activity, and the maintenance of correct body weight [1, 2]. Indeed, epidemiological evidence indicates that a poor quality diet, physical inactivity, and overweight and obesity are strong risk factors for multiple malignancies [3]. In this scenario, increasing numbers of foods and nutrients with a protective effect have been identified in recent years [4]. Despite the role of diet in cancer prevention, this evidence is widely perceived as inconsistent, underlining the need for greater research and communication clarity.

Cutaneous malignant melanoma (CMM) is the most dangerous form of skin cancer, having a growing incidence, high metastatic potential and affecting all age groups, which makes preventive measures particularly urgent. The incidence differs among countries but has increased markedly worldwide in recent years, especially in white-skinned populations [5]. CMM arises from melanocytes, the cells responsible for the production of the melanin pigment of the skin, hair, and eyes, and is the result of complex interactions between individual genetic factors and environmental risk factors. The scientific literature has provided direct evidence that sun exposure causes mutations in critical genes for melanoma [6]. Ultraviolet B (UVB) radiation is the most mutagenic component of the ultraviolet spectrum and promotes DNA damage more than ultraviolet A (UVA) radiation. UVB radiation is responsible for the production of DNA photoproducts such as cyclobutane pyrimidine dimers (CPDs). CPDs cause bulky lesions that distort the DNA helix, producing adducts that can suspend DNA replication and transcription. UVB can also damage DNA indirectly by causing oxidative stress resulting from lipid peroxidation and the formation of reactive oxygen and nitrogen intermediates [7, 8]. Moreover, exposure to UVB causes inflammation, including erythema and edema, and chronic inflammation is a recognized risk factor for tumor development [9].

Molecular pathways underlying melanoma genesis are complex; RAS-RAF-MEK-ERK mitogen-activated protein kinase (MAPK) and PI3K-PTEN-AKT (AKT) are the two major pathways constitutively activated through genetic abnormalities [10]. The two most common mutations occur in *BRAF* (40–55%) and *NRAS* (15–30%); clinically relevant *BRAF* mutations result in the substitution of valine at position 600 (*BRAF*^V600^) in the gene encoding BRAF serine-threonine kinase in the MAPK pathway [10].

**Fig. 1:**
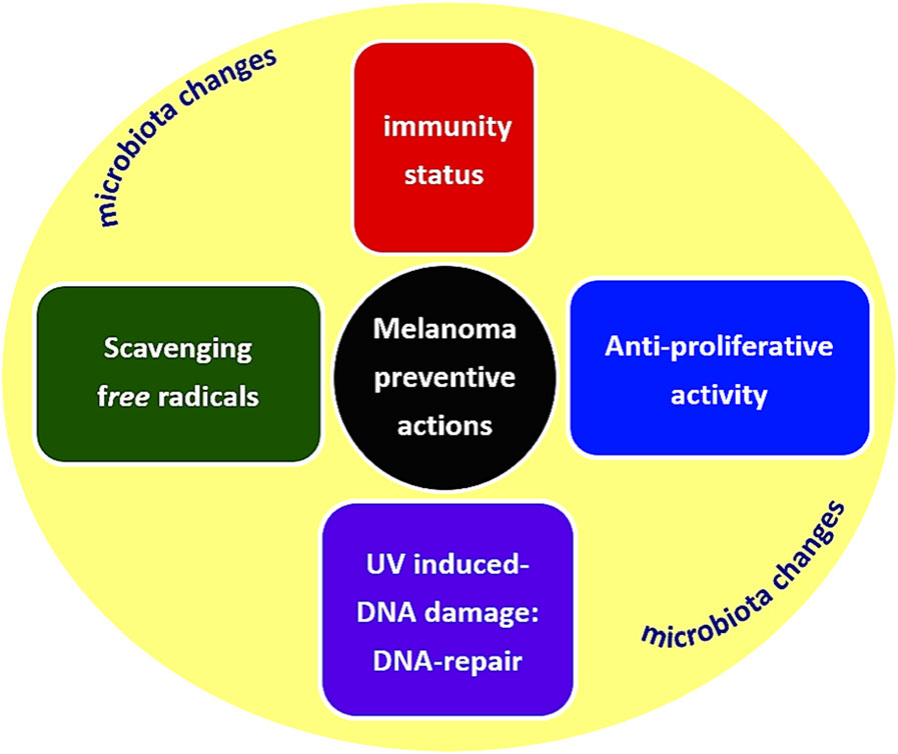
Extrinsic factors involved in melanoma prevention

As mentioned above, the incidence of CMM has continued to rise in recent years despite public efforts to promote sun protection habits. Considering that the use of sunscreen does not entirely prevent skin cancer, additional chemo-preventive approaches are desirable. In this regard, attention has been focused on the possible role of diet in reducing the melanoma risk. Furthermore, dietary interventions may have systemic benefits, unlike purely topical methods of sun protection, and do not need constant reapplication. Numerous studies have suggested a protective role of some dietary elements, but relationships between dietary intake of certain foods and the cancer risk are still controversial. Dietary antioxidant phytochemicals have demonstrated protective effects and the presence of these compounds in the traditional Mediterranean diet may be partly responsible for the low incidence of CMM in this area, despite high levels of solar radiation; other studies showed a trend towards a reduced risk of CMM with a greater intake of vegetables and fruit, fish, as well as vitamins and beverages such as coffee or tea [11, 12]. The results appear encouraging and could reinforce nutritional prevention campaigns and the development of appropriate initiatives. Extrinsic factors thought to play a role in melanoma prevention are summarized in Fig. 1.

In this review, we report the most recent advances on the comprehension of the biological mechanisms, which underlay the impact of foods and dietary compounds on the risk and prevention of melanoma. Considering the wideness of the topic, and in order to avoid redundancy, we chose to describe the main dietary compounds involved in active research with substantial advances in the last decade, excluding foods and compounds with well-known impact on the disease.

## Foods and melanoma risk: recent advances

**Fig. 2:**
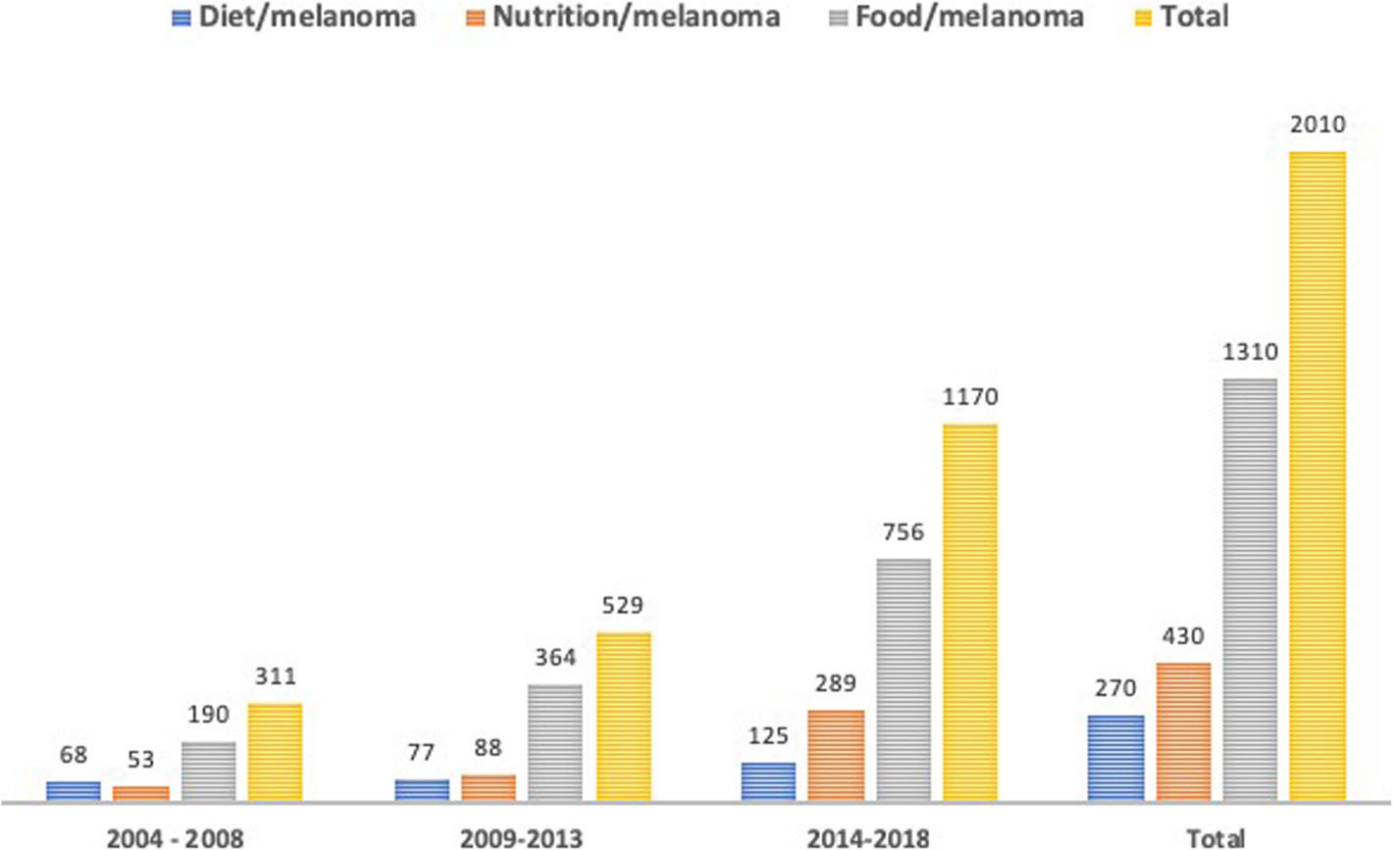
Total amount of manuscripts on nutrition and melanoma published in recent years in PubMed retrieved using the following keywords: “diet” or “nutrition” or “food” and “melanoma” (until the 31st December 2018)

There has been growing interest in the role of nutrition for melanoma prevention in recent years, as demonstrated by the rising of the total number of articles published in PubMed on the topic (Fig. 2). Numerous epidemiological studies have widely demonstrated that regular consumption of fruit and vegetables is associated with a reduced risk of cancer [13]. Modification of diet alone, by increasing vegetable and fruit intake, could even prevent cancer. This evidence has awakened interest in research on bioactive food components, and has led to the identification of compounds with a cancer preventive and therapeutic potential. Owing to their safety, low toxicity and antioxidant properties, fruits, vegetables and other dietary elements (phytochemicals and minerals) have being analysed as chemopreventive agents, intended to interrupt the carcinogenesis process, which includes the initiation, promotion, and progression of otherwise normal cells to cancer. Some evidence has also suggested that a variety of substances may enhance the therapeutic efficacy of drugs, reduce chemotherapy-induced side effects or overcome drug-resistance [14–16].

Bioactive food substances are identified on the basis of in vitro and in vivo studies. These compounds present tumor-suppressing properties in animal models of carcinogenesis, interfering with cellular processes of tumor formation. In phase II studies in humans, it has frequently been impossible to draw definite conclusions about the preventive or clinical efficacy because of the great variability and differences in study designs, patient numbers, study duration, as well as lack of a standardized formulation. Lastly, it is not always easy to reach a consensus due to discordant results obtained in similar studies.

In melanocytes, reactive oxygen species (ROS) accumulate - including singlet oxygen (^1^O_2_), hydrogen peroxide (H_2_O_2_), and superoxide (O_2_^−^) - leading to oxidative stress-induced cell damage. In general, ROS may induce antioxidant defenses by enhancing the expression of superoxide dismutase, catalase, glutathione peroxidase, and peroxiredoxins, which maintain the redox balance [17, 18]. However, when cellular ROS production overwhelms the antioxidant capacity, the ROS cause serious toxicity and damage in cells. Thus, ROS scavengers and inhibitors of ROS production may suppress melanomagenesis and protect against skin damage.

**Fig. 3:**
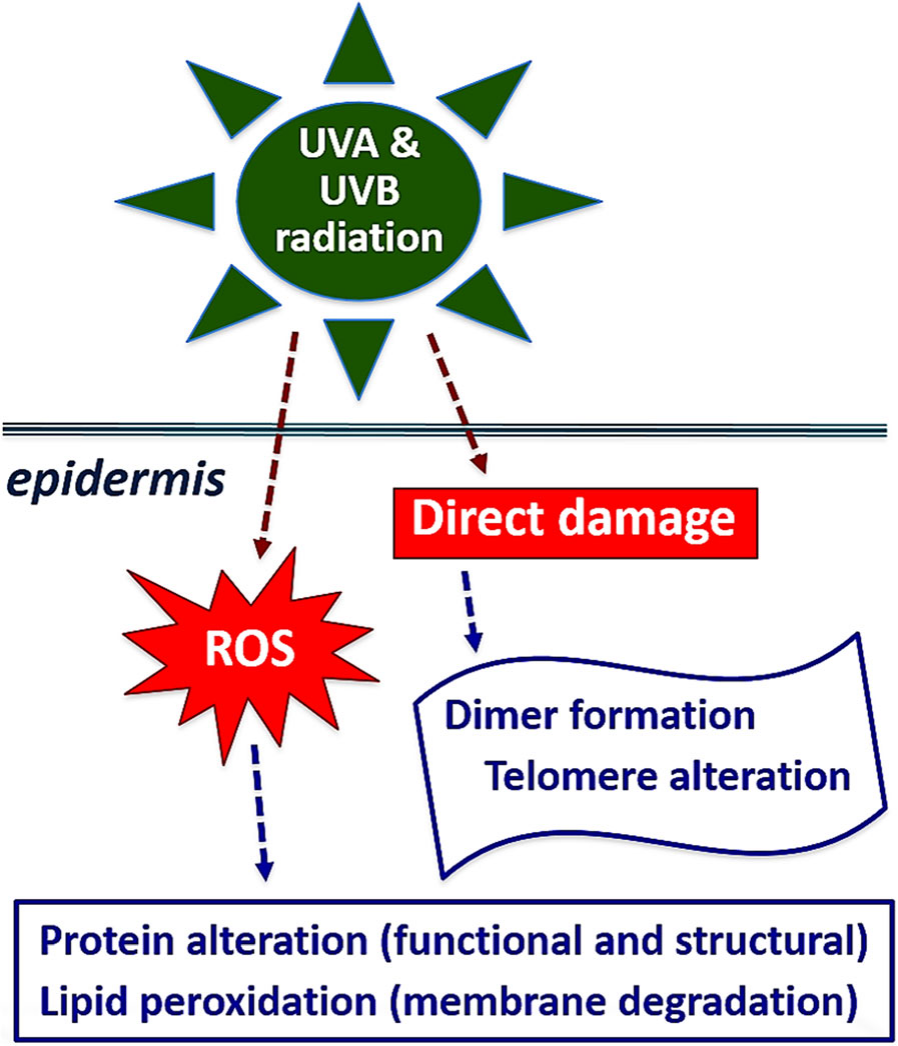
Main functional effects of UVA/B radiation on skin

From the molecular point of view, ROS are reported to activate p21ras protein through increased phosphorylation [19]. Another signaling molecule which has been shown to act as a direct target of ROS and nitrogen species is ataxia-telangiectasia mutated (ATM) protein kinase. It has been shown that the ATM protein is activated after certain stresses, most notably after double-stranded DNA breaks, through oxidation at the C-terminal region of ATM [20]. Moreover, cells carrying inactivated ATM exhibit constitutively high levels of ROS [21]. The mechanism whereby ATM regulates the intracellular redox state is complex and may involve alterations of some mTOR-dependent mechanisms [22]. In recent years, studies have shown that ROS activate COX (three isoforms of cyclooxygenase, namely COX1, COX2, and COX3) and that COX and its products induce ROS generation. A diagram of the main molecular effects triggered by ROS is shown in Fig. 3.

Natural antioxidants are a focus of skin protection due to their potential to scavenge ROS and inhibit the UV-induced signal transduction pathway, thus offering a promising strategy for combating melanogenesis [23, 24]. Many dietary compounds have been identified: vitamins, minerals, carotenoids and a large class of phytochemicals (polyphenols, isothiocyanates, organosulfur compounds), as well as sulforaphane, anthocyanidins, lycopene, diallyl disulfide, rosmarinic acid, silymarins, oleuropein, etc. [25, 26]. Both in vitro and in vivo studies have elucidated various cellular and molecular mechanisms by which such compounds scavenge ROS and act against melanoma cell formation; we will focus initially on coffee, tea and pomegranate, and then in specific dietary compounds in which consistent advances were carried out in the last decade.

Coffee and tea are the most widely consumed beverages worldwide. They contain numerous phytochemicals, many of which are antioxidants, such as chlorogenic acids, quinic acid, caffeic acid, ferulic acid, and coumaric acid among the polyphenols and caffeine, diterpenes (coffee lipids). The quantities of these components depend on the brewing method [27, 28], and were object of active research in relation to melanoma in recent years.

### Coffee

In vitro and animal studies suggest that bioactive constituents of coffee may have anti-carcinogenic effects against cutaneous melanoma; however, the epidemiological evidence is limited to date. Prospective studies on coffee consumption and malignant melanoma have shown conflicting results, ranging from no association to lower relative risk. Potential mechanisms of coffee phytochemicals include inhibition of oxidative stress and oxidative damage by ROS, regulation of DNA repair, phase II enzymatic activity, apoptosis, inflammation, as well as anti-proliferative, anti-angiogenetic effects, and antimetastatic effects.

According to Loftfield et al. [29], high coffee intake is associated with a lower risk of melanoma. The authors found a 20% lower risk for participants who drank 4 or more cups per day. The protective effect appeared to increase with a higher intake, increasing from 1 or fewer cups to 4 cups of coffee or more. The study on coffee consumption was performed on 447,357 white participants using a self-administered food frequency questionnaire in 1995 through 1996, and for a median follow-up of 10 years. The subjects were free of cancer at baseline and the authors adjusted for ultraviolet radiation exposure, body mass index, age, sex, physical activity level, alcohol intake, and smoking history. The preventive effect was found to be statistically significant only for caffeinated coffee, and only for protection against malignant melanoma and not melanoma in situ [29]. Their findings suggested that drinking four or more cups per day may decrease the risk of melanoma by 20%, but require replication also in other populations.

In another study, Wu et al. [30] reported that components in coffee and tea may have anti-carcinogenic properties. They prospectively analysed coffee, tea and CMM risk in the Women’s Health Initiative: a cohort study of 66,484 postmenopausal women, followed for an average of 7.7 years. Coffee and tea intakes were measured through self-administered questionnaires at the beginning and at year 3 of follow-up. Daily coffee and tea intakes were not significantly associated with melanoma risk compared with no-daily intake of each beverage. No significant trends were observed between melanoma risk and increasing intakes of coffee or tea. Women who reported a daily coffee intake at both the starting point and year 3 had a significantly decreased risk compared with women who reported non-daily intake at both time points (HR = 0.68, 95% CI 0.48–0.97). Daily tea intake was not associated with a decreased melanoma risk. They concluded that there is no strong evidence that increasing coffee or tea consumption can lead to lower melanoma risk [30].

.In another large study, Wu et al. [31] used data from 163,886 women in the Nurses’ Health Study II (NHS II, 1991–2009) and Nurses’ Health Study (NHS, 1980–2008) and 39,424 men in the Health Professionals Follow-up Study (HPFS, 1986–2008). They documented 2254 melanoma cases over 4 million person-years of follow-up. After adjustment for other risk factors, higher total caffeine intake was associated with a lower risk for CMM (≥393 mg/d vs. < 60 mg/d: HR = 0.78, 95% CI = 0.64–0.96, P trend = 0.048). The association was more apparent in women (≥393 mg/d vs. < 60 mg/d: HR = 0.70, 95% CI = 0.58–0.85, P trend = 0.001) than in men (HR = 0.94, 95% CI = 0.75–1.18, P trend = 0.81), and more apparent for melanomas occurring on body sites with a higher continuous sun exposure (head, neck and extremities) (≥393 mg/d vs. < 60 mg/d: HR = 0.71, 95% CI = 0.59–0.86, P trend = 0.001) than for melanomas on other body sites (trunk including shoulders, back, hips, abdomen and chest) (HR = 0.90, 95% CI = 0.70–1.16, P trend = 0.60). No association was found between decaffeinated coffee consumption and CMM risk. They concluded that caffeinated coffee consumption may be protective against CMM [31].

A meta-analysis of cohort studies was conducted by Wang et al. [32] to investigate the association between coffee and the most common cancer types. This study evidenced an inverse association between coffee intake and oropharyngeal cancer, liver cancer, colon cancer, prostate cancer, endometrial cancer, and melanoma but an increased association for lung cancer. The reduction was found to be up to 31% for oropharyngeal cancer, 13% for colon cancer, 54% for liver cancer, 11% for prostate cancer, 27% for endometrial cancer, and 11% for melanoma, for the highest compared to the lowest coffee intake [32]. Simultaneously, Wang et al. [33] conducted another meta-analysis to study the associations between the consumption of total coffee, caffeinated or decaffeinated coffees, and melanoma risk, respectively. They selected 12 studies including 832,956 participants for total coffee consumption, 5 studies involving 717,151 participants for caffeinated coffee consumption and 6 studies for a total of 718,231 participants for decaffeinated coffee consumption. This meta-analysis suggests that coffee consumption may reduce the risk of CMM. A dose-response analysis defined a decreased cutaneous melanoma risk by 3% [0.97 (0.93–1.00)] and 4% [0.96 (0.92–1.01)] per 1 cup/day increment of total coffee and caffeinated coffee consumption, respectively [33].

Also, Yew et al. [34] performed a meta-analysis of published studies to evaluate any association between coffee consumption and melanoma. Nine observational studies were identified, for a total of 927,173 participants, of which 3787 had melanoma. They calculated a 0.75 (95% CI 0.63–0.89, *p* = 0.001) relative risk (RR) for melanoma among regular coffee drinkers compared to controls. The pooled relative risk for melanoma among decaffeinated coffee drinkers was not, however, statistically significant, at 0.92 (95% CI 0.82–1.05, *p* = 0.215). The authors concluded that there is some evidence for a beneficial effect of regular coffee consumption on melanoma, but more studies would be necessary to confirm this association [34].

Liu et al. [35] identified and analyzed two case-control studies (846 CMM patients and 843 controls) and five cohort studies (including 844,246 participants and 5737 CMM cases). For caffeinated coffee, the RR for CMM was 0.81 (95% CI = 0.68–0.97; *P*-value for Q-test = 0.003; I2 = 63.5%) for those with the highest versus lowest quantity of coffee intake. In the dose-response analysis, the RR for CMM was 0.955 (95% CI = 0.912–0.999) per 1 cup/day increment of caffeinated coffee consumption, and a linear dose-response association was found (P-value = 0.326). Moreover, no significant association was found between the decaffeinated coffee intake and CMM risk (RR = 0.92; 95% CI = 0.81–1.05; P-value for Q-test = 0.967; I2 = 0%) for the highest versus lowest quantity of intake. This meta-analysis concluded that caffeinated coffee might have preventive actions against malignant melanoma but not decaffeinated coffee, in accordance with previous studies [35]. According to Lukic et al. [36] who performed the Norwegian Women and Cancer (NOWAC) study, moderate consumption of filtered coffee is associated with a decreased risk of malignant melanoma. Interestingly, the authors found no evidence of an association between instant, boiled, or total coffee consumption and the risk of CMM [35].

In a more recent study, Caini et al. [37] examined the relationships between coffee (total, caffeinated or decaffeinated) and tea consumption and the risk of melanoma in the European Prospective Investigation into Cancer and Nutrition (EPIC). EPIC was a multicentre prospective study that enrolled over 500,000 participants aged 25–70 years from ten European countries in the years 1992–2000. Information on coffee and tea drinking were collected at baseline using validated country-specific dietary questionnaires. In this study, 2712 melanoma cases were identified during a median follow-up of 14.9 years among 476,160 participants. Consumption of caffeinated coffee was inversely associated with melanoma risk among men (HR for the highest quartile of consumption versus non-consumers 0.31, 95% CI 0.14–0.69) but not among women (HR 0.96, 95% CI 0.62–1.47). There were no statistically significant associations between the consumption of decaffeinated coffee or tea and the melanoma risk among men or women. In this large cohort study, consumption of caffeinated coffee was inversely associated with melanoma risk, only among men [37].

**Fig. 4:**
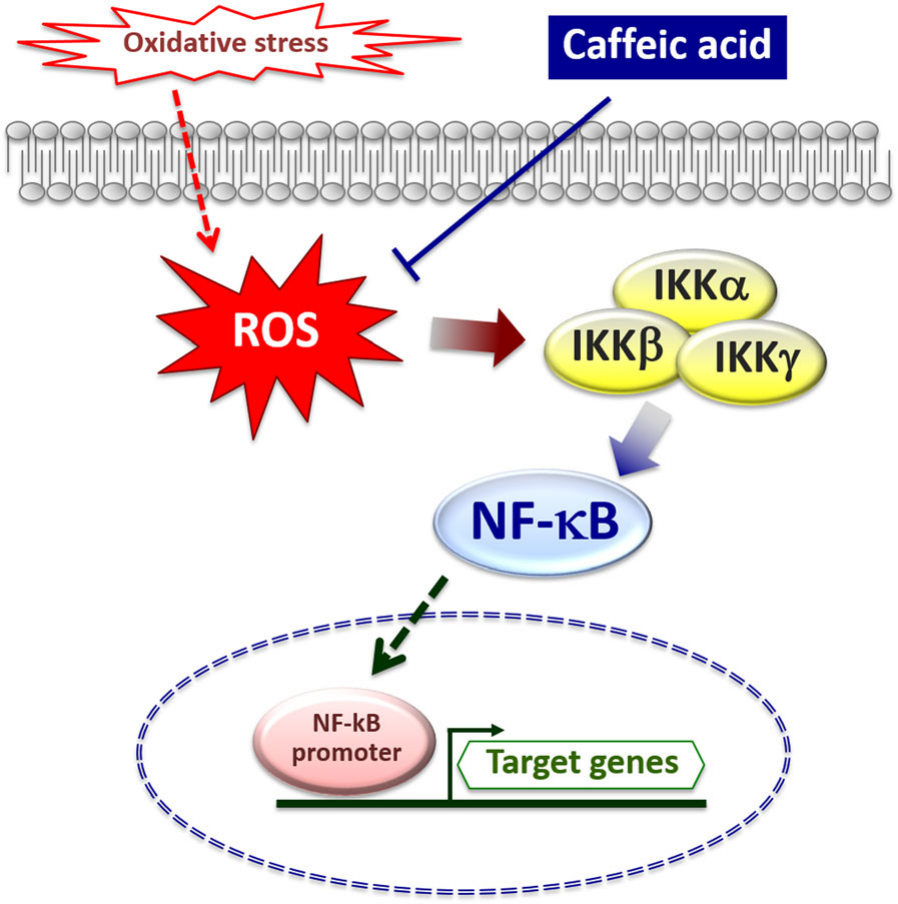
The activity of caffeic acid on the molecular mechanism controlling cell survival

A further study by Conney et al. [38] examined the effects of caffeine and the molecular mechanisms at the basis of its protective effect. They indicated that caffeine administration inhibits UVB-induced carcinogenesis by enhancing apoptosis in UVB-induced tumors. The stimulatory effect of caffeine on apoptosis occurs by p53-dependent and p53-independent mechanisms. Inhibition of the ATR/Chk1 pathway by caffeine is a major contributor to caffeine inhibition of UVB-induced carcinogenesis. In addition, a p53-independent effect indicated that caffeine enhanced UVB-induced apoptosis by inhibiting the increase in ATR-mediated formation of phospho-Chk1 (Ser345) and abolishing the decrease in cyclin B1, which resulted in caffeine-induced premature, lethal mitosis in mouse skin. In short, ATR-mediated phosphorylation of Chk1 is an important target for caffeine’s inhibitory effect on UVB-induced carcinogenesis. Moreover, caffeic acid inhibited the activation of the IKK-NF-κB signaling pathway by scavenging intracellular ROS generated by oxidative stress (Fig. 4). Upon activation, NF-kB can undergo retention in the nucleus of the cells and regulate the transcription of a wide variety of genes, including those involved in cell proliferation [39].

### Tea

Tea is also a popular beverage worldwide, derived from the infusion of leaves of *Camellia sinensis*, a species of the Theaceae family. The tea plant and its leaves have long been used for medicinal purposes. Several in vitro, in vivo, and epidemiological studies have reported that the consumption of green tea may decrease cancer risk. In particular, green tea and its major polyphenol constituents, such as epicatechin (EC), epicatechin-3-gallate (ECG), epigallocatechin (EGC), and epigallocatechin-3-gallate (EGCG), have been shown to possess many beneficial properties for health; furthermore, black tea polyphenols can induce apoptosis of melanoma cell lines in vitro [40]. Evidence is now accumulating that catechins and theaflavins, which are the main polyphenolic compounds of green and black tea, are responsible for further beneficial effects.

Regarding melanoma, large epidemiological studies did not evidence a strong association between the consumption of tea and risk prevention [30, 37]. Nevertheless, interesting results come from in vitro and in vivo studies. The anti-cancer properties of green tea are referred mainly to epigallocatechin-3-gallate (EGCG). Owing to its chemical properties, EGCG may act both as a sunscreen and as a quencher of free radicals [41]. Experiments in mouse models of melanoma indicated that EGCG inhibits the formation of lung metastases after tail vein injection of B16 melanoma cells [42], whereas topical application showed partial inhibition of skin papilloma growth in mice. Epigallocatechin-3-gallate helps to reverse damage caused by UV light, and drinking green tea has caused a decrease in UV-induced skin tumor incidence and size compared with controls. In mice, green tea polyphenols have also caused inhibition of UV-induced matrix metalloproteinase-2, − 3, − 7, and − 9 expression, involved in degradation of the basement membrane, preliminary to metastasis [43].

**Fig. 5:**
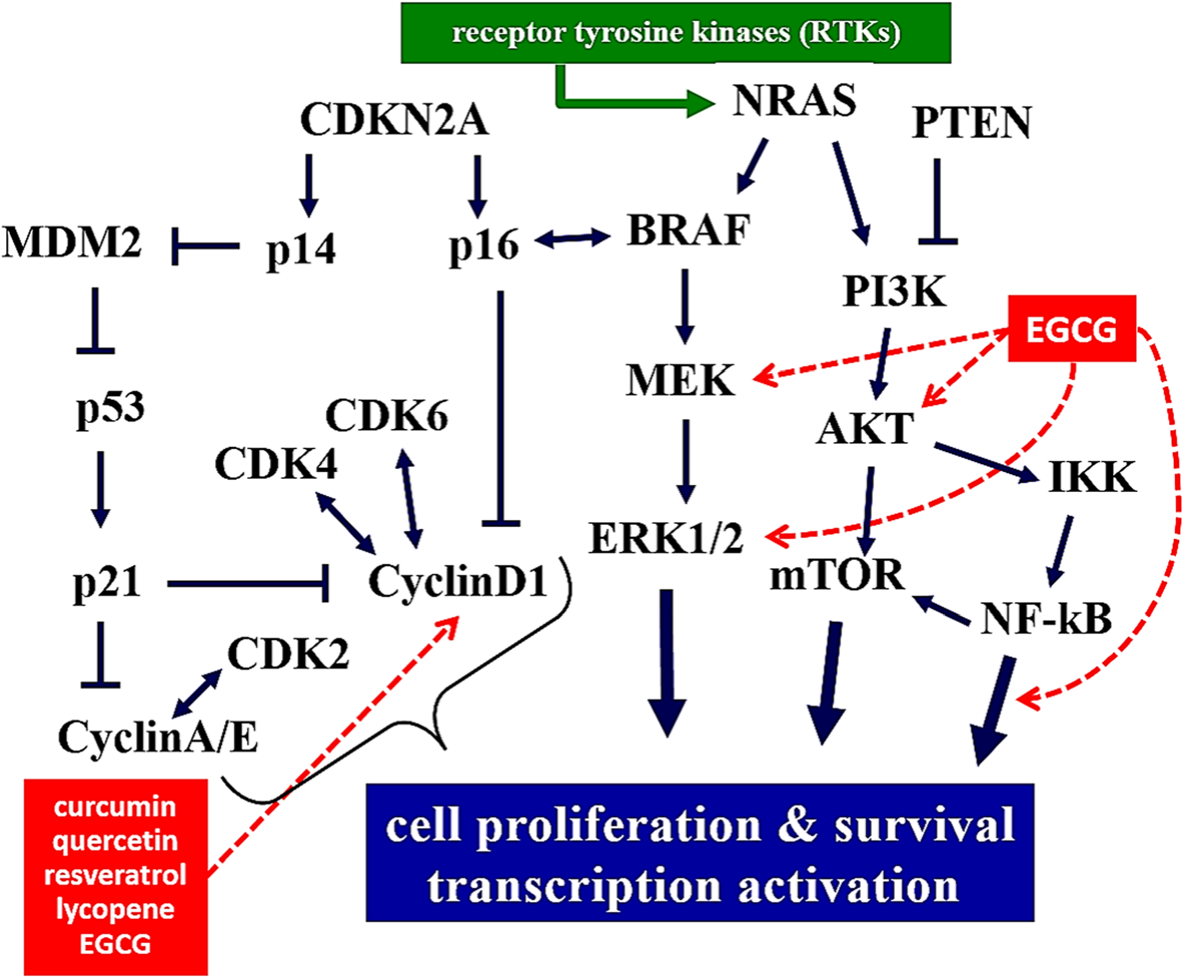
Dietary components interfering with the main molecular pathways of melanomagenesis

Animal studies have clearly demonstrated the anti-carcinogenic effects of EGCG through induction of melanoma cell apoptosis and cell cycle arrest by modulating B-cell lymphoma 2 (Bcl-2) and the CKI-Cyclin-CDK pathway [44–46]. In vitro studies evidenced that Green tea polyphenol epigallocatechin-3-O-gallate inhibits melanoma tumor growth by activating the 67-kDa laminin receptor (67LR) [44]. 67LR has been identified as a cell surface receptor of EGCG and plays a key role in the cancer preventive effects of EGCG. In melanoma, 67LR is expressed at a higher level than in normal skin cells. The authors have previously shown that EGCG suppresses melanoma tumor growth by activating the intercellular signaling pathway, cAMP/protein kinase A (PKA)/protein phosphatase 2A, as an agonist of 67LR. They assessed the involvement of 67LR signaling pathway in the miRNA regulation mechanism of EGCG. Tea polyphenols have also been implicated in multiple carcinogenesis pathways, including angiogenesis inhibition, immune system modulation, and activation of enzymatic systems involved in cellular detoxification through the glutathione S-transferase and quinone reductase pathways [45, 46]. Overall, EGCG, accounting for up to 80% of the total antioxidant polyphenols called catechins in tea, exert inhibitory effects on several components of the signaling cascades, which control proliferation and survival of cells of the melanocytic lineage (Fig. 5).

However, the concentrations of EGCG required to elicit the anticancer effects in a variety of cancer cell types are much higher than the peak plasma concentration registered after drinking an equivalent of 2–3 cups of green tea. Furthermore, the anti-cancer efficacy of EGCG can be due to or enhanced by combining it synergistically with other chemical compounds, mixtures of specific polyphenols or mixtures of polyphenols with vitamins, amino acids, and other micronutrients.

Previous human studies have demonstrated the topical effects of EGCG, which inhibits erythema, oxidative stress, and infiltration of inflammatory leukocytes and enhances pyrimidine dimer repair in DNA, in UV-irradiated human skin. Because of these properties, some skin-care products, including sunscreens, contain green tea extracts, although in many cases their quantitative polyphenol content is not standardized [47]. Still, the photoprotective bioactivities of orally administered polyphenols were validated in a 12-week, double-blind, placebo-controlled study [48]. Moreover, in a clinical study, topical use of 660 μM EGCG for 2 weeks during radiotherapy was non-toxic for patients with non-inflammatory breast cancer, effectively preventing radiation-induced dermatitis and significantly lowering the symptom scores of burning, pain, and itching [49]. Therefore, polyphenols and EGCG can relieve carcinogen-induced cutaneous damages and may then help to prevent cutaneous carcinogenesis.

The conflicting results obtained in the studies examined may be because of the various types of teas used, as well as variable tea preparations, unknown concentrations of different antioxidants, and also the bioavailability of many of these compounds after ingestion may be different across populations. Furthermore, many of these studies are often influenced by the intake of other protective or harmful substances, and it is difficult to distinguish these confounding variables [50]. Further preclinical and clinical studies on green tea compounds and, especially, polyphenols for the prevention of skin cancers including melanoma are required.

### Pomegranate

Several studies have demonstrated that pomegranates possess strong anti-oxidant actions due to their free radical scavenging capacity [51]. These fruits possess anti-proliferative, anti-inflammatory and anti-tumorigenic functions [52–54]. In preclinical animal studies, oral consumption of pomegranate extract inhibited the growth of lung, skin, colon and prostate tumors. Several clinical studies have been conducted on human volunteers. Pomegranate extract, given to 70 patients with diagnosed prostate adenocarcinoma for 4 weeks decreased 8-hydroxy-20-deoxyguanosine (8-OHdG), an oxidative stress biomarker. The presence of pomegranate metabolites was shown in benign and malignant prostate tissues [55]. Nevertheless, no epidemiological studies investigating their role in preventing melanoma are currently available.

Kang et al. [23] examined the underlying mechanisms of dried pomegranate concentrate powder (PCP) on melanin synthesis in B16F10 melanoma cells. Pomegranates are rich in ellagic acid and other polyphenols, such as flavonoids and hydrolyzable tannins. Recently, it was demonstrated that the skin-whitening effects of pomegranates are due to the inhibition of proliferation and melanin synthesis by tyrosinase in melanocytes. The results obtained in B16F10 cells suggest that pomegranate decreases tyrosinase activity and melanin production via inactivation of the p38 and PKA signaling pathways, and subsequently decreases phosphorylation of CREB, MITF, and melanogenic enzymes. Oral feeding of pomegranate fruit extract (PFE, 0.2%, wt/vol) was found to sustain protection from the adverse effects of single UVB radiation in mice. UVB-induced epidermal hyperplasia, infiltration of leukocytes, protein oxidation and lipid peroxidation were inhibited by pomegranate [56]. Pomegranate also elicited significant suppression of UVB-induced protein expression of COX-2, iNOS, PCNA, cyclin D1, and matrix metalloproteinases-2, − 3 and − 9. Moreover, the protection mechanism involved the inhibition of UVB-induced nuclear translocation and phosphorylation of NF-κB /p65, phosphorylation, and degradation of IκBα, activation of IKKα/IKKβ as well as phosphorylation of MAPK and c-Jun. [56]

In another study performed with HaCaT cell line, pomegranate seed oil nano-emulsion entrapping polyphenol-rich ethyl acetate fractions was able to protect the DNA against UVB-induced damage [57]. Studies in a mouse skin tumorigenesis model also showed that the combination of polyphenols and diallyl sulfide (DAS) synergistically reduced the tumor incidence by interfering with cell proliferation and by stimulating apoptosis, as shown by histological and cell death analysis [58]. In SKH-1 hairless mice, pomegranate fruit extract (PFE) reduced UVB-NF-κB activation and mitogen-stimulated protein kinase pathways. Per-oral administration of PFE (0.2%,wt/vol) for 14 days decreased the UVB-mediated skin edema, hyperplasia, infiltration of leukocytes, lipid peroxidation, hydrogen peroxide generation, ornithine decarboxylase (ODC) activity, expression of ODC, COX-2 and proliferating cell nuclear antigen protein. In addition, PFE increased the repair of UVB-stimulated production of cyclo-butane pyrimidine dimers and 8-oxodG. PFE increased the UVB-mediated rise of tumor suppressor p53 and cyclin kinase inhibitor p21. Per-oral administration of PFE reduced the nuclear translocation of NF-κB, activation of IKKα and phosphorylation and degradation of IκBα mediated by UVB [59].

In a mouse model, topical application of PFE 2 mg prior to the application of 3.2 nmole 12-O-tetradecanoyl phorbol-13-acetate (TPA) time-dependently elicited a significant inhibition of the TPA-induced rise in skin edema and hyperplasia, epidermal ODC activity and protein expression of ODC and COX-2 [60]. In addition, PFE showed a decrease of TPA-mediated phosphorylation of ERK1/2, p38 and JNK1/2, as well as the activation of NF-κB and IKKα, and phosphorylation and degradation of IKBα. PFE-treated animals revealed a reduced tumor incidence and lower tumor volume burden. All these studies indicate that PFE is a strong antitumoral agent in animal models. More clinical trials are required to confirm the efficacy of pomegranate [60].

## Nutrients/phytochemicals and melanoma

### Resveratrol

Resveratrol is a natural polyphenol commonly found in fruits, grape skins, mulberries, and red wine. Early basic research studies identified anticancer effects of resveratrol against several different tumors and in multiple stages of tumor initiation and proliferation [61]. Specifically, resveratrol can induce cancer cell apoptosis by interfering with multiple signaling pathways of the transformed cells. Resveratrol may also promote immune-surveillance through the innate immune system, thereby influencing the elimination of spontaneous tumor cells prior to proliferation [62].

To delineate this role, a clinical trial focused on detecting differences in immune system profiles was performed in healthy subjects given 1 g of resveratrol daily for 2 weeks. Preclinical studies had confirmed that resveratrol can induce the expression of NKG2D ligands in transformed cells and thus render these cells more susceptible to NK cell lysis via NKG2D cytotoxic pathways [63]. Resveratrol may modulate this axis to allow for increased tumor surveillance by the innate immune system. Moreover, it has previously been shown to protect human skin from the effects of sun damage by decreasing sunburn cell formation [64, 65]. Pharmacokinetic evidence indicates that resveratrol has poor bioavailability due to its rapid and extensive liver metabolism, which severely impairs its therapeutic effects. Melanoma cells often rely on alpha-melanocyte-stimulating hormone signal transduction, a crucial process in the development and spread of melanoma cells, that is suppressed by resveratrol [66, 67]. The alpha-melanocyte-stimulating hormone has also demonstrated immunosuppressive properties and beneficial effects in modulating chronic inflammation, by down-regulating major histocompatibility complex (MHC) molecules, in addition to CD40, CD80, and CD86 co-stimulatory molecules [68, 69]. Resveratrol was shown to have other anticancer properties; in particular, it exerts anti-proliferative activity against melanoma A431 cells and induces apoptosis in A475 and SK-mel28 cells [70, 71].

Although human studies are limited, further data have shown that resveratrol is pharmacologically safe, making it a prime candidate for potential future cancer therapeutic agents. Resveratrol may also be an effective adjuvant treatment, as it prevents endothelial cell injury in high-dose interleukin 2 therapy for melanoma. A topical application of a formulation containing 1% resveratrol, 0.5% baicalin and 1% vitamin E for 12 weeks can mildly modulate photo-damaged skin, improving the chances of cutaneous rejuvenation [72, 73]. Given the low bioavailability of this compound when administered either orally or topically, novel formulation strategies have been attempted. Researchers have designed dermal resveratrol delivery into human skin by using formulation techniques such as micro-emulsions [74] or lipid-core nano-capsules [75]. In addition, Amiot et al. developed a soluble resveratrol formulation that had an 8.8-fold higher plasma concentration in healthy volunteers than that of powders [76]. Based on these pharmaceutical achievements in human subjects, it seems necessary to further verify the chemo-preventive activities of resveratrol.

### Vitamins

Vitamin A (retinol) is a fat-soluble compound that is necessary for normal physiologic function and cannot be synthesized by humans, is therefore classified as an essential nutrient [77]. Vitamin A is obtained in the form of retinyl esters from the diet, mostly from animal sources such as eggs, milk, and liver. Also, plant-based pro-vitamin A carotenoids, such as α-carotene and β-cryptoxanthin, can be converted to vitamin A in the intestine but only < 10% of these carotenoids can undergo conversion [78, 79].

The effect of vitamin A on melanoma development is of particular interest. Results from epidemiologic studies concerning the association between vitamin A intake and melanoma risk are still controversial [80]. Older evidence suggests that retinoids have powerful effects in inhibiting cell growth, proliferation, inducing apoptosis and differentiation in human and murine melanoma cell lines. Dietary carotenoids have antioxidant properties, thus reducing the risk of UV-induced skin tumors in mice, and the administration of vitamin A has been proposed as a melanoma chemoprevention approach; pro-vitamin A carotenoids have also been proven to exert an anti-melanoma activity through alternate pathways including anti-angiogenic effects by altering cytokines profiles and nuclear translocation of transcription factors in melanoma cell lines [80–85].

Vitamin C may also have a potential role in melanoma chemoprevention [86, 87]. It is an essential water-soluble nutrient that acts as an antioxidant and a cofactor of various metabolic enzymes [88]. Moreover, vitamin C exerts effects on host defense mechanisms through the maintenance of immune homeostasis [89]. It has dual properties in oxidative processes, acting as an antioxidant and pro-oxidant in the presence of metal ions [90]. As an antioxidant, it protects cells and tissues from oxidative stress due to its conversion to the oxidized form, dehydroascorbic acid (DHA), that is reduced to ascorbic acid inside the cells, thereby decreasing intracellular ROS levels. On the other hand, it also accelerates oxidative metabolism by preventing the use of pyruvate for glycolysis. This feature helps to inhibit the proliferation of tumor cells, but not normal cells. Ascorbate decreases the mitochondrial membrane potential, activates caspase 3 which results in apoptosis in melanoma A375 cells. Ascorbate is even responsible for a decrease of HIF-1 levels, through the inhibition of COX-2 expression, through IGF-II production and caspase-independent autophagy [90–93].

Vitamin E and its various derivatives have demonstrated photo-protective and anti-oxidative properties against melanoma in animal studies. However, the results in epidemiological studies have been less convincing [94–96]. The studies have failed to demonstrate a clear relationship between the dietary intake of vitamin E and melanoma incidence. Accordingly, it has been suggested that oral supplementation may not have a clinically significant effect [62]. From a biological point of view, there are eight natural compounds that have been found to have vitamin E activity: D-α-, D-β-, D-γ- and D-δ-tocopherol, and D-α-, D-β-, D-γ- and D-δ-tocotrienol. α-Tocopherol may inhibit melanin synthesis both directly by inactivating tyrosinase, which is the key enzyme of melanogenesis in melanocytes, and by affecting the post-translation levels of tyrosinase-related protein 1 and 2 [97]. According to Kamei et al. [98], other forms of tocopherol (D-β-tocopherol and D-γ-tocopherol) have a promising anti-melanogenetic activity with less cytotoxicity at relatively high concentrations. Moreover, it has been reported that vitamin E succinate can inhibit the growth and survival of melanoma cells in vitro [99], while another study reported anti-melanoma effects of vitamin E succinate in vivo [100]. Vitamin E also reduces IL-6 and IFN-γ production by different leukocyte subsets and limits the toxic effects of ROS released during inflammation [101]. The translational value of these evidences remain to be clarified.

Vitamin D status has been widely suggested to affect cancer risk and play a role in cancer prevention (including melanoma) by exerting anti-proliferative effects [102, 103]. Solar radiation is critical for vitamin D synthesis in humans; however, uncontrolled and intensive sun exposure is dangerous to skin health and may contribute to the development of cutaneous malignant melanoma [104]. A correct balance between sun protection/exposure and vitamin D status is thus advocated. In recent years, there has been growing interest in understanding the link between vitamin D levels and melanoma**.** There are epidemiological studies to confirm the hypothesis that higher vitamin D levels might protect from melanoma, although a number of cohort studies have addressed a possible protective effect of vitamin D [103–108]. Nevertheless, there are insufficient indications to recommend vitamin D supplementation to decrease melanoma risk.

Vitamin D has a clear anti-proliferative activity on melanoma cell lines in vitro [109]. There is evidence of reduced expression of the vitamin D receptor during progression from nevi through primary to metastatic melanoma. These observations suggest that if vitamin D is anti-proliferative for melanoma cells in vivo, then those cells might be less likely to respond to the anti-proliferative effects of vitamin D as progression occurs. A high circulating vitamin D concentration has been found to be associated with reduced melanoma progression and improved survival. The reported effects of vitamin D on the immune system are extremely complex. If vitamin D supplements suppress adaptive immunity, then that would be a potentially harmful effect in melanoma patients. High doses of vitamin D are also to be avoided. The evidence that vitamin D levels might influence melanoma risk remains uncertain; however, it should also be pointed out that no studies of sufficient size to address this issue have been conducted [110]. In addition, patients with CMM who strictly avoid sun exposure might benefit from 25(OH) D supplements that are sufficient to maintain serum levels above 30 ng mL-1. Given the interest in using vitamin D to reduce cancer risk, more research is warranted to establish its role in the control and progression of melanoma, and whether vitamin D supplements can reduce cancer risk and progression and improve outcomes. Interestingly, it has been also shown that vitamin D could be used to control immune-related adverse events mediated by Th-17+ cell expansion occurring during immunotherapy for CMM [111–113].

### Flavonoids

Flavonoids are a large group of polyphenolic compounds (more than 5000) found in vegetables, which exhibit anti-tumor activities that are attracting more and more attention in chemoprevention and cancer treatment. The molecular mechanisms of flavonoids and their activities in antioxidant, anti-inflammation and immune modulation, anti-proliferation, anti-angiogenesis, apoptosis induction, and epigenetic modifications have been studied in vitro, or in mouse [114]. Large epidemiological studies (including melanoma) are currently lacking. The molecular mechanisms of flavonoids as antioxidants can be summarized in three major categories:

- Reacting directly with free radicals via their free hydroxyl group(s) and quenching these activities.
- As chelators for redox-potent transition metal ions, Cd2+,Fe2+, Co 2+, Ni 2+, Cu 2+, Cr 3+ and Zn2+ [46, 47]. These metals cause a ROS increase and the metal binding sites for flavonoids are usually adjacent to hydroxyl and/or ketone side groups.
- Modulating multiple cellular anti-oxidant systems which re-establish the redox balance in cells after oxidative stress.

Flavonoids modulate inflammatory effects through a few key mediators in melanoma and skin tissues: AP-1 [115], NFkB [116], STAT3 [117] and nitric oxidases (mainly iNOS and nNOS) [118, 119]. Flavonoids exhibit also anti-proliferative and anti-apoptotic effects via HGF/SF-Met signaling, MAPK pathway and PI3K-Akt pathway [120].

#### Proanthocyanidins

Proanthocyanidins are effective antioxidants and anti-inflammatory agents found in particularly high concentrations in grapes (GSPs) [60]. GSPs have been found to reduce UV skin damage, like photo-aging, and to decrease melanin synthesis [121, 122]. .In humans, GSPs have been shown to reduce mutant p53-positive epidermal cells and prevent the depletion of Langerhans cells after sunburns. Mouse studies have also yielded strong evidence supporting the inhibition of UV-induced tumor incidence, growth, and size, as well as metastatic pulmonary nodules, after the administration of grape seed extract [123, 124]. GSPs were also shown to inhibit cell migration in highly metastasis-specific human A375 and Hs294t melanoma cell lines: 22 to 65%, (*P* < 0.01) and 29 to 69%, (P < 0.01), respectively. In addition, GSPs decreased tissue plasminogen activator-induced activation of extracellular-signal-regulated kinase 1/2 protein and nuclear factor-κB/p65. These proteins have been shown to enhance and mediate the migration of melanoma cells. The inhibitory effects of GSPs on NF-κB also helped to reverse the epithelial-to-mesenchymal transition occurring in both melanoma cell lines. This evidence suggests a potential utilization as an anti-melanoma agent, considering that no toxicity has been shown in vivo [125].

#### Luteolin

Luteolin is another flavonoid common to many plants. It protects against SSBs (single-strand breaks) induced by oxidative stress in PC12 rat pheochromocytoma cells [126]. It possessed apoptotic potential in human lung squamous carcinoma CH27 cells, showing greater DNA damage and “S” phase cell cycle arrest [127]. Luteolin activates intrinsic apoptotic pathways by inducing DNA damage and p53 in many cancer cells [128, 129]. It induced apoptosis by inhibiting fatty acid synthase, a key lipogenic enzyme overexpressed in many human cancers [130]. Moreover. a chemo-preventive effect of luteolin and associated mechanisms were reported in the JB6 P+ neoplastic mouse cell line and the SKH-1 hairless mouse models [131]. Luteolin has been shown to delay or block the development of cancer cells both in vitro and in vivo, to protect DNA and induce cell cycle arrest and apoptosis via intrinsic and extrinsic signaling pathways [132]. Additionally, luteolin induces apoptosis in multidrug-resistant cancer cells by ROS generation, DNA damage initiation, activation of ATR/Chk2/p53 signaling, inhibition of NF-kB signaling, activation of p38, and depletion of anti-apoptotic proteins [133]. In addition, luteolin inhibits the hypoxia-induced epithelial-mesenchymal transition (EMT) in malignant melanoma cells both in vitro and in vivo via the regulation of β3 integrin [134]. Another study demonstrated that luteolin 7-sulfate isolated from *P. iwatensis* (a marine plant) is a human TYR inhibitor with advantageous anti-melanogenic properties, and would be a useful agent for the control of unwanted skin pigmentation [135].

#### Apigenin

Like luteolin, apigenin is a natural dietary flavonoid with anti-inflammatory and anti-oxidant properties. Epidemiological evidence suggests that apigenin intake reduces the risk of cancers and it has been found that apigenin inhibited ultraviolet light-induced skin carcinogenesis in mice. Subsequent studies also suggested anti-melanoma effects of apigenin, including inhibition of melanoma metastasis [136, 137]. In Cao [138], the involvement of the STAT3 signaling pathway in the anti-metastatic effect of apigenin was examined. Two human melanoma cell lines, A375 and G361, with constitutive activation of STAT3, together with a murine melanoma cell line, B16F10, were employed, showing that inhibition of the STAT3 signaling pathway contributes to the anti-metastatic effect of apigenin. In view of the reported anti-proliferative activity and low toxicity property of this compound, apigenin may also have a potential role in melanoma treatment or prevention. In Table 1, the anti-melanoma effects of the main dietary compounds are synthesized.

**Table 1:** Dietary compounds and their effects against melanoma

Dietary source/compounds	Anti-melanoma effect	References
Coffee/various phytochemicals	inhibition of oxidative stress and oxidative damage, regulation of DNA repair, phase II enzymatic activity, apoptosis, inflammation, antiproliferative, antiangiogenetic effects, and antimetastatic effects	29–39
Tea/catechins and theaflavins	reverse damage caused by UV light; decrease in UV-induced skin tumor incidence and size inhibiting angiogenesis, modulation of the immune system; activation of enzyme systems involved in cellular detoxification; EGCG inhibits erythema, enhances pyrimidine dimer repair in DNA, in UV-irradiated human skin	40–50
Pomegranate	decreases tyrosinase activity and melanin production; decreases phosphorylation of CREB, MITF, and melanogenic enzymes; strong antitumor agent in animal models	51–50
Resveratrol	antiproliferative activity against melanoma cells, induction of apoptosis; modulation of photodamaged skin	61–76
Vitamin A	Inhibition of growth, proliferation, apoptosis-induction, alteration of cytokines profiles	77–85
Vitamin C	to limit the toxic effects of ROS, immune homeostasis, apoptosis	86–93
Vitamin D	anti-proliferative activity, effects on the immune system	109–113
Vitamin E	reduction of IL-6 and IFN-γ production by different leukocyte subset, to limit the toxic effects of ROS, tyrosinase-inactivation	94–101
Flavonoids: GSPs, Luteolin, Apigenin, etc.	protection against UV damage; Induction of apoptosis Inhibition of cell growth in cell lines. Reversed epithelial-to-mesenchymal transition	114–138

### Dietary lipids

Several studies suggest that high dietary fat intake is directly associated with the risk of colorectal, liver, breast, pancreatic, gastrointestinal and prostate cancer [139, 140]. An increased intake of certain fatty acids promotes cancer growth while some other fatty acids have shown protective roles against cancer incidence. For example, palmitic acid and stearic acid seem to be potentially mutagenic to colonocytes [141], while the intake of arachidonic acid is not associated with colorectal cancer risk [142]. Dietary intake of linoleic acid increases the risk of prostate cancer; while consumption of ω-3 polyunsaturated fatty acids, docosahexaenoic and eicosapentaenoic acid, is associated with a decreased incidence of prostate cancer [143].

In a recent epidemiological study performed by Donat – Vargas et al. the authors controlled for sun habits and skin type, including 20,785 women from the prospective population-based Swedish Mammography Cohort. Validated estimates of dietary PCB exposure and eicosapentaenoic acid-docosahexaenoic acid (EPA-DHA) intake were obtained via a food frequency questionnaire. They ascertained 67 cases of melanoma through register-linkage. After multivariable adjustments, exposure to dietary PCBs was associated with a four-fold increased risk of malignant melanoma (HR 4.0, 95% CI 1.2–13; P for trend = 0.02]), while EPA-DHA intake was associated with an 80% lower risk (HR 0.2, 95% CI 0.1–0.8; P for trend = 0.03), when comparing the highest exposure tertiles with the lowest. While a direct association between dietary PCB exposure and the risk of melanoma exists, EPA-DHA intake was shown to have a substantial protective association.

Although the effects of different dietary fatty acids on cancer pathogenicity are diverse, it is generally believed that an excessive intake of certain fatty acids or the development of obesity and complications caused by the excess calorie intake promotes cancer growth [144].

Another aspect to consider is metastasis. Recently, a small population of CD36+ cells, that are highly predisposed to promote metastasis and are predominantly defined by a lipid metabolism signature, has been identified [145]. Pascual et al. described a subpopulation of CD44 bright cells in human oral carcinomas that do not overexpress mesenchymal genes, are slow-cycling, express high levels of the fatty acid receptor CD36 and lipid metabolism genes, and are unique in their ability to initiate metastasis. Palmitic acid or a high-fat diet specifically boost the metastatic potential of CD36+ metastasis-initiating cells in a CD36-dependent manner [145]. Two recent studies evidenced that ω-3 polyunsaturated fatty acids exert antitumourigenic activities against melanoma metastasis, via autophagy-mediated p62 elimination, CXCR4 suppression, and anti-inflammatory properties [146, 147].

### PCB and melanoma risk

Other than ultraviolet (UV) radiation risk factors may play a role in melanoma-genesis, such as environmental chemical exposures [148]. Polychlorinated biphenyls (PCBs) are synthetic organochlorine chemicals with well-described toxicity [149]. PCBs, which are widespread in the environment, accumulating in the food chain (they are classified in Group 1 as carcinogenic to humans by the International Agency for Research on Cancer) [150]. People are exposed to PCBs primarily through food, in particular when eating fatty fish. PCBs are absorbed and accumulated in adipose tissue, with a half-life ranging from 2 to 10 years [151].

The study of Donat-Vargas mentioned above is the only epidemiological study reporting results on interactions of PCBs and melanoma [152]. Gallagher et al. [153] conducted a case-control study of 80 CMM patients and 310 controls, collecting sun exposure information, data on pigmentation and sun sensitivity, along with a blood sample from each. Cases and controls were assayed for plasma levels of 14 PCB congeners and 11 organochlorine pesticide residues using gas chromatography. Strong associations were seen between the risk of CMM and plasma levels of non-dioxin-like PCBs (adjusted OR = 7.02; 95% CI: 2.30–21.43) and several PCB congeners, organochlorine pesticides or metabolites. These associations persisted after controlling for sun sensitivity and sun exposure.

## Conclusions

A great number of studies have been published recently investigating the roles of several dietary compounds in the prevention, development, and therapy of melanoma. Several foods and nutrients have been shown to have protective effects against melanoma-genesis or synergic effects with the medications used for CMM treatment. Recent literature reviews and data from the World Cancer Research Fund describe the epidemiological aspects of the interactions between diet and melanoma [154, 155]. In the present review, we focused mainly on recent advances regarding the biological mechanisms which lay under such interactions, involving specific compounds of current active research. Numerous encouraging results emerged, alone with conflicting outcomes, especially when basic research data are transferred to humans. This may depend on the heterogeneity of the compounds studied, their concentration, preparation, and administration, as well as on the heterogeneity of the methodological approaches and laboratory techniques employed. Further studies, at both the basic research and epidemiological level, performed with standardized approaches are needed to better comprehend the value of a wide range of nutrients in the prevention and clinical management of melanoma.

## Abbreviations

8-OHdG: 8-hydroxy-20-deoxyguanosine
ATM: Ataxia-telangiectasia mutated
CI: Confidence intervals
CMM: Cutaneous malignant melanoima
COX: Cyclooxygenase
CPDs: Cyclobutane pyrimidine dimers
DAS: Diallyl sulphide
DHA: Dehydroascorbic acid
EC: Epicatechin
ECG: Epicatechin-3-gallate
EGC: Epigallocatechin
EGCG: Epigallocatechin-3-gallate
EMT: Epithelial-mesenchymal transition
EPA-DHA: Eicosapentaenoic acid-docosahexaenoic acid
EPIC: European prospective investigation into cancer and nutrition
GSPs: Grapes proanthocyanidins
HPFS: Health professionals’ follow-up study
HR: Hazard ratio
MHC: Major histocompatibility complex
NHS: Nurses’ health study
ODC: Ornithine decarboxylase
PCBs: Polychlorinated biphenyls
PCP: Pomegranate concentrate powder
PFE: Pomegranate fruit extract
PUFAs: Polyunsaturated fatty acids
ROS: Reactive oxygen species
RR: Relative risk
SSB: Single-strand break
TPA: O-tetradecanoyl phorbol-13-acetate
UVA: Ultraviolet A
UVB: Ultraviolet B

## References

[1] Allen K (2018). The importance of food, nutrition and physical activity in cancer prevention: an interview with Dr Kate Allen. Future Oncol.

[2] Schuz J, Espina C, Villain P, Herrero R, Leon ME, Minozzi S (2015). European code against cancer 4th edition: 12 ways to reduce your cancer risk. Cancer Epidemiol.

[3] Vineis P, Wild CP (2014). Global cancer patterns: causes and prevention. Lancet.

[4] Zanini S, Marzotto M, Giovinazzo F, Bassi C, Bellavite P (2015). Effects of dietary components on cancer of the digestive system. Crit Rev Food Sci Nutr.

[5] Cossu A, Casula M, Cesaraccio R, Lissia A, Colombino M, Sini MC (2017). Epidemiology and genetic susceptibility of malignant melanoma in North Sardinia, Italy. Eur J Cancer Prev.

[6] Sini MC, Doneddu V, Paliogiannis P, Casula M, Colombino M, Manca A (2018). Genetic alterations in main candidate genes during melanoma progression. Oncotarget..

[7] Budden T, Bowden NA (2013). The role of altered nucleotide excision repair and UVB-induced DNA damage in melanomagenesis. Int J Mol Sci.

[8] Mazouzi A, Vigouroux A, Aikeshev B, Brooks PJ, Saparbaev MK, Morera S (2013). Insight into mechanisms of 3′-5′ exonuclease activity and removal of bulky 8,5′-cyclopurine adducts by apurinic/apyrimidinic endonucleases. Proc Natl Acad Sci U S A.

[9] Garssen J, Van Loveren H (2001). Effects of ultraviolet exposure on the immune system. Crit Rev Immunol.

[10] Palmieri G, Ombra M, Colombino M, Casula M, Sini M, Manca A (2015). Multiple molecular pathways in melanomagenesis: characterization of therapeutic targets. Front Oncol.

[11] Forsea AM, Del Marmol V, de Vries E, Bailey EE, Geller AC (2012). Melanoma incidence and mortality in Europe: new estimates, persistent disparities. Br J Dermatol.

[12] Malagoli C, Malavolti M, Agnoli C, Crespi CM, Fiorentini C, Farnetani F (2015). Diet quality and risk of melanoma in an italian population. J Nutr.

[13] Arts IC, Hollman PC (2005). Polyphenols and disease risk in epidemiologic studies. Am J Clin Nutr.

[14] Rengarajan T, Yaacob NS (2016). The flavonoid fisetin as an anticancer agent targeting the growth signaling pathways. Eur J Pharmacol.

[15] Amin AR, Kucuk O, Khuri FR, Shin DM (2009). Perspectives for cancer prevention with natural compounds. J Clin Oncol.

[16] Surh YJ (2003). Cancer chemoprevention with dietary phytochemicals. Nat Rev Cancer.

[17] Halliwell B (2011). Free radicals and antioxidants – quo vadis?. Trends Pharmacol Sci.

[18] Denat L, Kadekaro AL, Marrot L, Leachman SA, Abdel-Malek Z (2014). Melanocytes as instigators and victims of oxidative stress. J Invest Dermatol..

[19] Brennan LA, Wedgwood S, Bekker JM, Black SM (2003). Nitric oxide activates p21ras and leads to the inhibition of endothelial NO synthase by protein nitration. DNA Cell Biol.

[20] Guo Z, Kozlov S, Lavin MF, Person MD, Paull TT (2010). ATM activation by oxidative stress. Science..

[21] Reichenbach J, Schubert R, Schindler D, Müller K, Böhles H, Zielen S (2002). Elevated oxidative stress in patients with ataxia telangiectasia. Antioxid Redox Signal.

[22] Alexander A, Cai SL, Kim J, Nanez A, Sahin M, MacLean KH (2010). ATM signals to TSC2 in the cytoplasm to regulate mTORC1 in response to ROS. PNAS.

[23] Kang SJ, Choi BR, Lee EK, Kim SH, Yi HY, Park HR (2015). Inhibitory effect of dried pomegranate concentration powder on melanogenesis in B16F10 melanoma cells; involvement of p38 and PKA signaling pathways. Int J Mol Sci.

[24] Zhang H, Tsao R (2016). Dietary polyphenols, oxidative stress, and antioxidant and anti-inflammatory effects. Curr Opin Food Sci.

[25] Hu S, Zhang X, Chen F, Wang M (2017). Dietary polyphenols as photoprotective agents against UV radiation. J Funct Foods.

[26] Chaiprasongsuk A, Onkoksoong T, Pluemsamran T, Limsaengurai S, Panich U (2016). Photoprotection by dietary phenolics against melanogenesis induced by UVA through Nrf2-dependent antioxidant responses. Redox Biol.

[27] Kang NJ, Lee KW, Shin BJ, Jung SK, Hwang MK, Bode AM (2009). Caffeic acid, a phenolic phytochemical in coffee, directly inhibits Fyn kinase activity and UVB-induced COX-2 expression. Carcinogenesis..

[28] Lee KA, Chae JI, Shim JH (2012). Natural diterpenes from coffee, cafestol, and kahweol induce apoptosis through regulation of specificity protein 1 expression in human malignant pleural mesothelioma. J Biomed Sci.

[29] Loftfield E, Freedman ND, Graubard BI, Hollenbeck AR, Shebl FM, Mayne ST, et al. Coffee drinking and cutaneous melanoma risk in the NIH-AARP diet and health study. J Natl Cancer Inst. 2015. 10.1093/jnci/dju421. 10.1093/jnci/dju421 PMC4311176 25604135

[30] Wu H, Reeves KW, Qian J, Sturgeon SR (2015). Coffee, tea, and melanoma risk among postmenopausal women. Eur J Cancer Prev.

[31] Wu S, Han J, Song F, Cho E, Gao X, Hunter DJ, Qureshi AA (2015). Caffeine intake, coffee consumption, and risk of cutaneous malignant melanoma. Epidemiology..

[32] Wang A, Wang S, Zhu C, Huang H, Wu L, Wan X (2016). Coffee, and cancer risk: a meta-analysis of prospective observational studies. Sci Rep.

[33] Wang J, Li X, Zhang D (2016). Coffee consumption and the risk of cutaneous melanoma: a meta-analysis. Eur J Nutr.

[34] Yew YW, Lai YC, Schwartz RA (2016). Coffee consumption and melanoma: a systematic review and meta-analysis of observational studies. Am J of Clin Dermatol.

[35] Liu J, Shen B, Shi M, Cai J (2016). Higher caffeinated coffee intake is associated with reduced malignant melanoma risk: a meta-analysis study. PLoS One.

[36] Lukic M, Jared M, Weiderpass E, Braaten T (2016). Coffee consumption and the risk of malignant melanoma in the Norwegian women and Cancer (NOWAC) study. BMC Cancer.

[37] Caini S, Masala G, Saieva C, Kvaskoff M, Savoye I, Sacerdote C (2017). Coffee, tea and melanoma risk: findings from the European prospective investigation into Cancer and nutrition. Int J Cancer.

[38] Conney AH, Lu YP, Lou YR, Kawasumi M, Nghiem P (2013). Mechanisms of caffeine-induced inhibition of UVB carcinogenesis. Front Oncol.

[39] Dhawan P, Singh AB, Ellis DL, Richmond A (2002). Constitutive activation Akt/protein kinase B in melanoma leads to up-regulation of nuclear factor-kB and tumor progression. Cancer Res.

[40] Halder B, Bhattacharya U, Mukhopadhyay S, Giri AK (2008). Molecular mechanism of black tea polyphenols induced apoptosis in human skin cancer cells: involvement of Bax translocation and mitochondria mediated death cascade. Carcinogenesis..

[41] Nichols JA, Katyar SK (2010). Skin photoprotection and natural polyphenol:antiinflammatory, antioxidant and DNA repair mechanisms. Arch Dermatol Res.

[42] Taniguchi S, Fujiki H, Kobayashi H, Go H, Miyado K, Sadano H (1992). Effect of (−)-epigallocatechin gallate, the main constituent of green tea, on lung metastasis with mouse B16 melanoma cell lines. Cancer Lett.

[43] Vayalil PK, Mittal A, Hara Y, Elmets CA, Katiyar SK (2004). Green tea polyphenols prevent ultraviolet light-induced oxidative damage and matrix metalloproteinases expression in mouse skin. J Invest Dermatol.

[44] Yamada S, Tsukamoto S, Huang Y, Makio A, Kumazoe M, Yamashita S (2016). Epigallocatechin-3-O-gallate up-regulates microRNA-let-7b expression by activating 67-kDa laminin receptor signaling in melanoma cells. Sci Rep.

[45] Kotecha R, Takami A, Espinoza JL (2016). Dietary phytochemicals and cancer chemoprevention: a review of the clinical evidence. Oncotarget..

[46] Zhang J, Lei Z, Huang Z, Zhang X, Zhou Y, Luo Z (2016). Epigallocatechin-3-gallate (EGCG) suppresses melanoma cell growth and metastasis by targeting TRAF6 activity. Oncotarget..

[47] Grimaldi AM, Cassidy PB, Leachmann S, Ascierto PA (2014). Novel approaches in melanoma prevention and therapy. Cancer Treat Res.

[48] Heinrich U, Moore CE, De Spirt S, Tronnier H, Stahl W (2011). Green tea polyphenols provide photoprotection, increase microcirculation, and modulate skin properties of women. J Nutr.

[49] Zhu W, Jia L, Chen G, Zhao H, Sun X, Meng X (2016). Epigallocatechin-3-gallate ameliorates radiation-induced acute skin damage in breast cancer patients undergoing adjuvant radiotherapy. Oncotarget..

[50] Vuong QV (2014). Epidemiological evidence linking tea consumption to human health: a review. Crit Rev Food Sci Nutr.

[51] Turrini E, Ferruzzi L, Fimognari C (2015). Potential effects of pomegranate polyphenols in cancer prevention and therapy. Oxidative Med Cell Longev.

[52] Afaq F, Saleem M, Krueger CG, Reed JD, Mukhtar H (2005). Anthocyanin- and hydrolyzable tannin-rich pomegranate cancer fruit extract modulates MAPK and NF-κB pathways and inhibits skin tumorigenesis in CD-1 mice. Int J Cancer.

[53] Wang RF, Xie WD, Zhang Z, Xing DM, Ding Y, Wang W (2004). Bioactive compounds from the seeds of Punica granatum (pomegranate). J Nat Prod.

[54] Yoshimura M, Watanabe Y, Kasai K, Yamakoshi J, Koga T (2005). Inhibitory effect of an ellagic acid-rich pomegranate extract on tyrosinase activity and ultraviolet-induced pigmentation. Biosci Biotechnol Biochem.

[55] Freedland SJ, Carducci M, Kroeger N, Partin A, Rao JY, Jin Y (2013). A double-blind, randomized, neoadjuvant study of the tissue effects of POMx pills in men with prostate cancer before radical prostatectomy. Cancer Prev Res (Phila).

[56] Panth N, Manandhar B, Paudel KR (2017). Anticancer activity of Punica granatum (pomegranate): a review. Phytother Res.

[57] Baccarin T, Mitjans M, Lemos-Senna E, Vinardell MP (2015). Protection against oxidative damage in human erythrocytes and preliminary photosafety assessment of Punica granatum seed oil nanoemulsions entrapping polyphenol-rich ethyl acetate fraction. Toxicol in Vitro.

[58] George J, Singh M, Srivastava AK, Bhui K, Shukla Y (2011). Synergistic growth inhibition of mouse skin tumors by pomegranate fruit extract and diallyl sulfide: evidence for inhibition of activated MAPKs/NF-κB and reduced cell proliferation. Food Chem Toxicol.

[59] Afaq F, Khan N, Syed DN, Mukhtar H (2010). Oral feeding of pomegranate fruit extract inhibits early biomarkers of UVB radiation-induced carcinogenesis in SKH-1 hairless mouse epidermis. Photochem Photobiol.

[60] Parrado C, Philips N, Gilaberte Y, Juarranz A, González S. Oral Photoprotection: Effective Agents and Potential Candidates. Front Med (Lausanne). 2018;5:188. 10.3389/fmed.2018.00188 PMC6028556 29998107

[61] Savouret JF, Quesne M (2002). Resveratrol and cancer: a review. Biomed Pharmacother.

[62] Tong LX, Young LC (2014). Nutrition: the future of melanoma prevention?. J Am Acad Dermatol.

[63] Luis Espinoza J, Takami A, Trung LQ, Nakao S (2013). Ataxia-telangiectasia mutated kinase-mediated upregulation of NKG2D ligands on leukemia cells by resveratrol results in enhanced natural killer cell susceptibility. Cancer Sci.

[64] Reagan-Shaw S, Afaq F, Aziz MH (2004). Modulations of critical cell cycle regulatory events during chemoprevention of ultraviolet B-mediated responses by resveratrol in SKH-1 hairless mouse skin. Oncogene..

[65] Wu Y, Jia LL, Zheng YN, Xu XG, Luo YJ, Wang B (2013). Resveratrate protects human skin from damage due to repetitive ultraviolet irradiation. J Eur Acad Dermatol Venereol.

[66] Sim DY, Sohng JK, Jung HJ (2016). Anticancer activity of 7,8-dihydroxyflavone in melanoma cells via downregulation of α-MSH/cAMP/MITF pathway. Oncol Rep.

[67] Chen YJ, Chen YY, Lin YF, Hu HY, Liao HF (2013). Resveratrol inhibits alpha-melanocyte-stimulating hormone signaling, viability, and invasiveness in melanoma cells. Evid Based Complement Alternat Med.

[68] Aziz MH, Reagan-Shaw S, Wu J, Longley BJ, Ahmad N (2005). Chemoprevention of skin cancer by grape constituent resveratrol: relevance to human disease?. FASEB J.

[69] Svajger U, Obermajer N, Jeras M (2010). Dendritic cells treated with resveratrol during differentiation from monocytes gain substantial tolerogenic properties upon activation. Immunology..

[70] Niles RM, McFarland M, Weimer MB, Redkar A, Fu YM, Meadows GG (2003). Resveratrol is a potent inducer of apoptosis in human melanoma cells. Cancer Lett.

[71] Larrosa M, Tomás-Barberán FA, Espín JC (2003). Grape polyphenol resveratrol and the related molecule 4-hydroxystilbene induce growth inhibition, apoptosis, S-phase arrest, and upregulation of cyclins a, E, and B1 in human SK-Mel-28 melanoma cells. J Agric Food Chem.

[72] Wang S, Shen P, Zhou J, Lu Y (2017). Diet phytochemicals and cutaneous carcinoma chemoprevention: a review. Pharmacol Res.

[73] Farris P, Yatskayer M, Chen N, Krol Y, Oresajo C (2014). Evaluation of efficacy and tolerance of a nighttime topical antioxidant containing resveratrol, baicalin, and vitamin e for treatment of mild to moderately photodamaged skin. J Drugs Dermatol.

[74] Juškaitė V, Ramanauskienė K, Briedis V (2015). Design and formulation of optimized microemulsions for dermal delivery of resveratrol. Evid Based Complement Alternat Med.

[75] Friedrich RB, Kann B, Coradini K, Offerhaus HL, Beck RC, Windbergs M (2015). Skin penetration behavior of lipid-core nanocapsules for simultaneous delivery of resveratrol and curcumin. Eur J Pharm Sci.

[76] Amiot MJ, Romier B, Dao TM, Fanciullino R, Ciccolini J, Burcelin R (2013). Optimization of trans-resveratrol bioavailability for human therapy. Biochimie..

[77] Tanumihardjo SA (2011). Vitamin a: biomarkers of nutrition for development. Am J Clin Nutr.

[78] Tanumihardjo SA, Russell RM, Stephensen CB, Gannon BM, Craft NE, Haskell MJ (2016). Biomarkers of nutrition for development (BOND)-vitamin a review. J Nutr.

[79] Van Berkel TJ (2009). Bringing retinoid metabolism into the 21st century. J Lipid Res.

[80] Asgari MM, Brasky TM, White E (2012). Association of vitamin a and carotenoid intake with melanoma risk in a large prospective cohort. J Invest Dermatol..

[81] Estler M, Boskovic G, Denvir J, Miles S, Primerano DA, Niles RM (2008). Global analysis of gene expression changes during retinoic acid-induced growth arrest and differentiation of melanoma: comparison to differentially expressed genes in melanocytes vs melanoma. BMC Genomics.

[82] Luke JJ, Triozzi PL, McKenna KC, Van Meir EG, Gershenwald JE, Bastian BC (2015). Biology of advanced uveal melanoma and next steps for clinical therapeutics. Pigment Cell Melanoma Res.

[83] Wang Z, Coleman DJ, Bajaj G, Liang X, Ganguli-Indra G, Indra AK (2011). RXRα ablation in epidermal keratinocytes enhances UVR-induced DNA damage, apoptosis, and proliferation of keratinocytes and melanocytes. J Invest Dermatol..

[84] Niles RM (2003). Vitamin a (retinoids) regulation of mouse melanoma growth and differentiation. J Nutr.

[85] Zhang YP, Chu RX, Liu H (2014). Vitamin a intake and risk of melanoma: a meta-analysis. PLoS One.

[86] Tremante E, Santarelli L, Lo Monaco E, Sampaoli C, Ingegnere T, Guerrieri R, Tomasetti M, Giacomini P. Sub-apoptotic dosages of pro-oxidant vitamin cocktails sensitize human melanoma cells to NK cell lysis. Oncotarget. 2015;6(31):31039-49 10.18632/oncotarget.5024 PMC4741587 26427039

[87] Yun J, Mullarky E, Lu C, Bosch KN, Kavalier A, Rivera K (2015). Vitamin C selectively kills KRAS and BRAF mutant colorectal cancer cells by targeting GAPDH. Science..

[88] Mamede AC, Tavares SD, Abrantes AM, Trindade J, Maia JM, Botelho MF (2011). The role of vitamins in cancer: a review. Nutr Cancer.

[89] Lin SY, Lai WW, Chou CC, Kuo HM, Li TM, Chung JG (2006). Sodium ascorbate inhibits growth via the induction of cell cycle arrest and apoptosis in human malignant melanoma A375.S2 cells. Melanoma Res.

[90] Kim HW, Cho SI, Bae S, Kim H, Kim Y, Hwang YI (2012). Vitamin C up-regulates expression of CD80, CD86 and MHC class II on dendritic cell line DC-1 via the activation of p38 MAPK. Immune Netw.

[91] Frömberg A, Gutsch D, Schulze D, Vollbracht C, Weiss G, Czubayko F (2011). Ascorbate exerts anti-proliferative effects through cell cycle inhibition and sensitizes tumor cells towards cytostatic drugs. Cancer Chemother Pharmacol.

[92] Kim HN, Kim H, Kong JM, Bae S, Kim YS, Lee N (2011). Vitamin C down-regulates VEGF production in B16F10 murine melanoma cells via the suppression of p42/44 MAPK activation. J Cell Biochem.

[93] Jensen JD, Wing GJ, Dellavalle RP (2010). Nutrition and melanoma prevention. Clin Dermatol.

[94] Anstey AV (2002). Systemic photoprotection with alpha-tocopherol (vitamin E) and beta-carotene. Clin Exp Dermatol.

[95] Klein EA, Thompson IM, Lippman SM, Goodman PJ, Albanes D, Taylor PR (2003). SELECT: the selenium and vitamin E Cancer prevention trial. Urol Oncol.

[96] Russo I, Caroppo F, Alaibac M (2015). Vitamins and melanoma. Cancers.

[97] Solano F, Briganti S, Picardo M, Ghanem G (2006). Hypopigmenting agents: an updated review on biological, chemical and clinical aspects. Pigment Cell Res.

[98] Kamei Y, Otsuka Y, Abe K (2009). Comparison of the inhibitory effects of vitamin E analogues on melanogenesis in mouse B16 melanoma cells. Cytotechnology..

[99] Kogure K, Manabe S, Suzuki I, Tokumura A, Fukuzawa K (2005). Cytotoxicity of alpha-tocopheryl succinate, malonate and oxalate in normal and cancer cells in vitro and their anti-cancer effects on mouse melanoma in vivo. J Nutr Sci Vitaminol (Tokyo).

[100] Malafa MP, Fokum FD, Mowlavi A, Abusief M, King M (2002). Vitamin E inhibits melanoma growth in mice. Surgery..

[101] Novoselova EG, Lunin SM, Novoselova TV, Khrenov MO, Glushkova OV, Avkhacheva NV (2009). Naturally occurring antioxidant nutrients reduce inflammatory response in mice. Eur J Pharmacol.

[102] Holick MF (2004). Sunlight and vitamin D for bone health and prevention of autoimmune diseases, cancers, and cardiovascular disease. Am J Clin Nutr.

[103] Cattaruzza Maria Sofia, Pisani Daniela, Fidanza Laura, Gandini Sara, Marmo Giovanna, Narcisi Alessandra, Bartolazzi Armando, Carlesimo Marta (2019). 25-Hydroxyvitamin D serum levels and melanoma risk. European Journal of Cancer Prevention.

[104] Gandini S, Montella M, Ayala F, Benedetto L, Rossi CR, Vecchiato A (2016). Sun exposure and melanoma prognostic factors. Oncol Lett.

[105] Nürnberg B, Gräber S, Gärtner B, Geisel J, Pföhler C, Schadendorf D (2009). Reduced serum 25-hydroxyvitamin D levels in stage IV melanoma patients. Anticancer Res.

[106] Newton-Bishop JA, Beswick S, Randerson- Moor J, Chang YM, Affleck P, Elliott F (2009). Serum 25-hydroxyvitamin D3 levels are associated with Breslow thickness at presentation and survival from melanoma. J Clin Oncol.

[107] Tagliabue E, Raimondi S, Gandini S (2015). Meta-analysis of vitamin D-binding protein and cancer risk. Cancer Epidemiol Biomark Prev.

[108] Fang S, Sui D, Wang Y, Liu H, Chiang YJ, Ross MI (2016). Association of Vitamin D levels with outcome in patients with melanoma after adjustment for C-reactive protein. J Clin Oncol.

[109] Ishibashi M, Arai M, Tanaka S, Onda K, Hirano T (2012). Antiproliferative and apoptosis-inducing effects of lipophilic vitamins on human melanoma A375 cells in vitro. Biol Pharm Bull.

[110] Ombra MN, Paliogiannis P, Doneddu V, Sini MC, Colombino M, Rozzo C (2017). Vitamin D status and risk for malignant cutaneous melanoma: recent advances. Eur J Cancer Prev.

[111] Sotirchos ES, Bhargava P, Eckstein C, Van Haren K, Baynes M, Ntranos A (2016). Safety and immunologic effects of high- vs low-dose cholecalciferol in multiple sclerosis. Neurology.

[112] Danlos FX, Pagès C, Roux J, Jebali M, Gornet JM, Bagot M (2015). Atypical severe immune-related adverse effects resulting from sequenced immunotherapy in melanoma. Melanoma Res.

[113] Stucci LS, D’Oronzo S, Tucci M, Macerollo A, Ribero S, Spagnolo F (2018). Vitamin D in melanoma: controversies and potential role in combination with immune check-point inhibitors. Cancer Treat Rev.

[114] George VC, Dellaire G, Rupasinghe HPV (2017). Plant flavonoids in cancer chemoprevention: role in genome stability. J Nutr Biochem.

[115] Schonthaler HB, Guinea-Viniegra J, Wagner EF (2011). Targeting inflammation by modulating the Jun/AP-1 pathway. Ann Rheum Dis.

[116] McNulty SE, del Rosario R, Cen D, Meyskens FL, Yang S (2004). Comparative expression of NFkappaB proteins in melanocytes of normal skin vs. benign intradermal naevus and human metastatic melanoma biopsies. Pigment Cell Res.

[117] Flashner-Abramson E, Klein S, Mullin G, Shoshan E, Song R, Shir A (2016). Targeting melanoma with NT157 by blocking Stat3 and IGF1R signaling. Oncogene..

[118] Grimm EA, Sikora AG, Ekmekcioglu S (2013). Molecular pathways: inflammation-associated nitric-oxide production as a cancer-supporting redox mechanism and a potential therapeutic target. Clin Cancer Res.

[119] Yang Z, Misner B, Ji H, Poulos TL, Silverman RB, Meyskens FL (2013). Targeting nitric oxide signaling with nNOS inhibitors as a novel strategy for the therapy and prevention of human melanoma. Antioxid Redox Signal.

[120] Liu-Smith F, Meyskens FL (2016). Molecular mechanisms of flavonoids in melanin synthesis and the potential for the prevention and treatment of melanoma. Mol Nutr Food Res.

[121] Cho HS, Kwak DH, Choi IS, Park HK, Kang SJ, Yoo HS (2009). Inhibitory effect of proanthocyanidin on ultraviolet B irradiation-induced melanogenesis. J Toxicol Environ Health A.

[122] Yuan XY, Liu W, Hao JC, Gu WJ, Zhao YS (2012). Topical grape seed proanthocyandin extract reduces sunburn cells and mutant p53 positive epidermal cell formation, and prevents depletion of Langerhans cells in an acute sunburn model. Photomed Laser Surg.

[123] Vaid M, Singh T, Prasad R, Katiyar SK (2016). Bioactive proanthocyanidins inhibit growth and induce apoptosis in human melanoma cells by decreasing the accumulation of β-catenin. Int J Oncol.

[124] Yamakoshi J, Otsuka F, Sano A, Tokutake S, Saito M, Kikuchi M (2003). Lightening effect on ultraviolet-induced pigmentation of Guinea pig skin by oral administration of a proanthocyanidin-rich extract from grape seeds. Pigment Cell Res.

[125] Katiyar Santosh K., Pal Harish C., Prasad Ram (2017). Dietary proanthocyanidins prevent ultraviolet radiation-induced non-melanoma skin cancer through enhanced repair of damaged DNA-dependent activation of immune sensitivity. Seminars in Cancer Biology.

[126] Silva JP, Gomes AC, Coutinho OP (2008). Oxidative DNA damage protection and repair by polyphenolic compounds in PC12 cells. Eur J Pharmacol.

[127] Leung HW, Wu CH, Lin CH, Lee HZ (2005). Luteolin induced DNA damage leading to human lung squamous carcinoma CH27 cell apoptosis. Eur J Pharmacol.

[128] Shi R, Huang Q, Zhu X, Ong YB, Zhao B, Lu J (2007). Luteolin sensitizes the anticancer effect of cisplatin via c-Jun NH2-terminal kinase-mediated p53 phosphorylation and stabilization. Mol Cancer Ther.

[129] George VC, Naveen Kumar DR, Suresh PK, Kumar S, Kumar RA (2013). Comparative studies to evaluate relative in vitro potency of luteolin in inducing cell cycle arrest and apoptosis in HaCaT and A375 cells. Asian Pac J Cancer Prev.

[130] Brusselmans K, Vrolix R, Verhoeven G, Swinnen JV (2005). Induction of cancer cell apoptosis by flavonoids is associated with their ability to inhibit fatty acid synthase activity. J Biol Chem.

[131] Byun S, Lee KW, Jung SK, Lee EJ, Hwang MK, Lim SH (2010). Luteolin inhibits protein kinase C (epsilon) and c-Src activities and UVB-induced skin cancer. Cancer Res.

[132] Seelinger G, Merfort I, Wölfle U, Schempp CM (2008). Anti-carcinogenic effects of the flavonoid luteolin. Molecules..

[133] Rao PS, Satelli A, Moridani M, Jenkins M, Rao US (2012). Luteolin induces apoptosis in multidrug resistant cancer cells without affecting the drug transporter function: involvement of cell line-specific apoptotic mechanisms. Int J Cancer.

[134] Ruan JS, Liu YP, Zhang L, Yan LG, Fan FT, Shen CS (2012). Luteolin reduces the invasive potential of malignant melanoma cells by targeting β3 integrin and the epithelial-mesenchymal transition. Acta Pharmacol Sin.

[135] Kwak JY, Seok JK, Suh HJ, Choi YH, Hong SS, Kim DS (2016). Antimelanogenic effects of luteolin 7-sulfate isolated from Phyllospadix iwatensis Makino. Br J Dermatol.

[136] Caltagirone S, Rossi C, Poggi A, Ranelletti FO, Natali PG, Brunetti M (2000). Flavonoids apigenin and quercetin inhibit melanoma growth and metastatic potential. Int J Cancer.

[137] Piantelli M, Rossi C, Iezzi M, La Sorda R, Iacobelli S, Alberti S (2006). Flavonoids inhibit melanoma lung metastasis by impairing tumor cells endothelium interactions. J Cell Physiol.

[138] Cao HH, Chu JH, Kwan HY, Su T, Yu H, Cheng CY (2016). Inhibition of the STAT3 signaling pathway contributes to apigenin-mediated anti-metastatic effect in melanoma. Sci Rep.

[139] Hursting SD, Thornquist M, Henderson MM (1990). Types of dietary fat and the incidence of cancer at five sites. Prev Med.

[140] Donaldson MS (2004). Nutrition and cancer: a review of the evidence for an anti-cancer diet. Nutr J.

[141] Beeharry N, Lowe JE, Hernandez AR, Chambers JA, Fucassi F, Cragg PJ (2003). Linoleic acid and antioxidants protect against DNA damage and apoptosis induced by palmitic acid. Mutat Res.

[142] Sakai M, Kakutani S, Horikawa C, Tokuda H, Kawashima H, Shibata H (2012). Arachidonic acid and cancer risk: a systematic review of observational studies. BMC Cancer.

[143] Pelser C, Mondul AM, Hollenbeck AR, Park Y (2013). Dietary fat, fatty acids, and risk of prostate cancer in the NIH-AARP diet and health study. Cancer Epidemiol Biomark Prev.

[144] Dossus L, Kaaks R (2008). Nutrition, metabolic factors and cancer risk. Best Pract Res Clin Endocrinol Metab.

[145] Pascual G, Avgustinova A, Mejetta S, Martín M, Castellanos A, Attolini CS (2017). Targeting metastasis-initiating cells through the fatty acid receptor CD36. Nature..

[146] Tan RH, Wang F, Fan CL, Zhang XH, Zhao JS, Zhang JJ (2018). Algal oil rich in n-3 polyunsaturated fatty acids suppresses B16F10 melanoma lung metastasis by autophagy induction. Food Funct.

[147] Li J, Chen CY, Arita M, Kim K, Li X, Zhang H, Kang JX (2018). An omega-3 polyunsaturated fatty acid derivative, 18-HEPE, protects against CXCR4-associated melanoma metastasis. Carcinogenesis..

[148] Berwick M, Buller DB, Cust A, Gallagher R, Lee TK, Meyskens F (2016). Melanoma epidemiology and prevention. Cancer Treat Res.

[149] Carpenter DO (2006). Polychlorinated biphenyls (PCBs): routes of exposure and effects on human health. Rev Environ Health.

[150] Lauby-Secretan B, Loomis D, Grosse Y, El Ghissassi F, Bouvard V, Benbrahim-Tallaa L (2013). Carcinogenicity of polychlorinated biphenyls and polybrominated biphenyls. Lancet Oncol.

[151] Milbrath MO, Wenger Y, Chang CW, Emond C, Garabrant D, Gillespie BW (2009). Apparent half-lives of dioxins, furans, and polychlorinated biphenyls as a function of age, body fat, smoking status, and breast-feeding. Environ Health Perspect.

[152] Donat-Vargas C, Berglund M, Glynn A, Wolk A, Åkesson A (2017). Dietary polychlorinated biphenyls, long-chain n-3 polyunsaturated fatty acids and incidence of malignant melanoma. Eur J Cancer.

[153] Gallagher RP, Macarthur AC, Lee TK, Weber JP, Leblanc A, Mark Elwood J (2011). Plasma levels of polychlorinated biphenyls and risk of cutaneous malignant melanoma: a preliminary study. Int J Cancer.

[154] Yang K, Fung TT, Nan H (2018). An epidemiological review of diet and cutaneous malignant melanoma. Cancer Epidemiol Biomark Prev.

[155] World Cancer Research Fund. How diet, nutrition and physical activity affect skin cancer. Available at: https://www.wcrf.org/dietandcancer/skin-cancer [accessed 16 Apr 2019].

